# Generating synthetic images of human skeletal motion for pose and kinematics estimation tasks

**DOI:** 10.1038/s41597-025-06243-7

**Published:** 2025-12-17

**Authors:** Jere Lavikainen, David Pagnon, Mimmi K. Liukkonen, Rami K. Korhonen, Mikael J. Turunen, Lauri Stenroth, Mika E. Mononen

**Affiliations:** 1 Department of Technical Physics, University of Eastern Finland, Kuopio, Finland; 2 Diagnostic Imaging Center, Kuopio University Hospital, Wellbeing Services County of North Savo, Kuopio, Finland; 3 Centre for the Analysis of Motion, Entertainment Research and Applications (CAMERA), University of Bath, Bath, United Kingdom; 4 Clinical Radiology, Kuopio University Hospital, Wellbeing Services County of North Savo, Kuopio, Finland; 5 Science Service Center, Kuopio University Hospital, Wellbeing Services County of North Savo, Kuopio, Finland

**Keywords:** Skeleton, Scientific data, Software

## Abstract

We developed a software that generates images of human motion from kinematics calculated using musculoskeletal modelling. The images are automatically annotated with information from the underlying skeletal model, including 3D positions of joint centers. The software enables the generation of an arbitrary number of images from a small number of skeletal poses by varying visual factors such as camera angle, background, body morphology, and skin and clothing textures of the person. The generation of synthetic images can be helpful in generating training data for supervised learning-based human pose estimation and motion tracking models. Because our software uses information from biomechanical models of the human musculoskeletal system, its annotations have the potential to be more accurate than those of existing large datasets of real images, where non-experts have marked the positions of anatomical landmarks. Additionally, new annotation points can be defined by editing the virtual marker set of the musculoskeletal model, which allows the generation of images with user-defined annotations.

## Background & Summary

Supervised learning methods, especially those relying on convolutional neural networks, have been applied in human motion analysis in recent years^1–14^. Their promise of tracking anatomical landmarks in images could make human motion analysis much more affordable and accessible than the current gold standard of optical motion capture^1^, which requires expensive equipment and post-processing^15^. After training on a dataset of images annotated with ground truth anatomical landmarks, human pose estimation (HPE) models could then be used to track anatomical landmarks in images (i.e., keypoints) using single or multiple video cameras.

Training HPE models requires tens of thousands of annotated images. Applicable existing datasets of real images include the Common Object in Context (COCO)^16^ and MPII Human Pose^17^ datasets, where people without anatomical expertise have often annotated the images. Because HPE models learn to detect those approximate annotations, inaccuracies in the annotated keypoints are also present in the final trained models. Inaccurate keypoint detection can be a problem when these HPE models are used in biomechanical studies of human motion, where accurate estimates of kinematics are desired^1–3,7^ and errors in kinematics can propagate in further analysis stages that require the derivation of kinematics^18–20^. Additionally, real datasets have limited coverage of condition- or motion-specific images, so the trained HPE models may perform poorly in tracking motion in those specific cases.

The generation of synthetic images offers a solution to the drawbacks of real images. If images are created artificially, the image and motion conditions can potentially be controlled, e.g., by generating images of specific motion types, which could alleviate the current need for motion type-specific HPE models^3^. Images can also be annotated automatically, which further eliminates much manual labour and ensures that there is no variance in the annotator-specific bias in the dataset.

Existing studies have shown that synthetic images are suitable for training accurate HPE models^21,22^. Furthermore, those studies have used anatomically accurate human models^23^ to annotate the images. While these are significant steps ahead, the synthetic images would be even more useful in training HPE models for human motion analysis if their annotations came directly from the virtual markers that can be, e.g., placed on the joint centers of the anatomical human model. When estimating the kinematics of motion with musculoskeletal (MSK) models, HPE models trained with such data could be used for analyzing video recordings to track keypoints that could replace motion capture markers – albeit with better matching between the tracked markers and virtual markers defined in the MSK model. While at least one study has developed a method for integrating MSK models in realistic human meshes, their “OpenSim-Driven Animated Human” dataset^24^ or the code to create it does not appear to be publicly available.

Here, we present a dataset of synthetic images with anatomically informed annotations and present our workflow for generating such images. Our workflow annotates images directly from information in MSK models, including the positions of virtual markers, and poses the visual human avatar in the generated images using kinematics calculated with the MSK modelling and simulation software OpenSim^25^. We hope that this integrated approach with MSK modelling makes the generated dataset particularly useful for HPE tasks that are followed by MSK modelling of human motion. We publicly share our image generation software and a sample dataset of generated images.

## Methods

### Overview

We first describe the workflow of the developed software to provide its working principles. Then, in the last subsection of methods (“**Dataset generation**”), we describe the protocol we used for generating the published data.

**Fig. 1:**
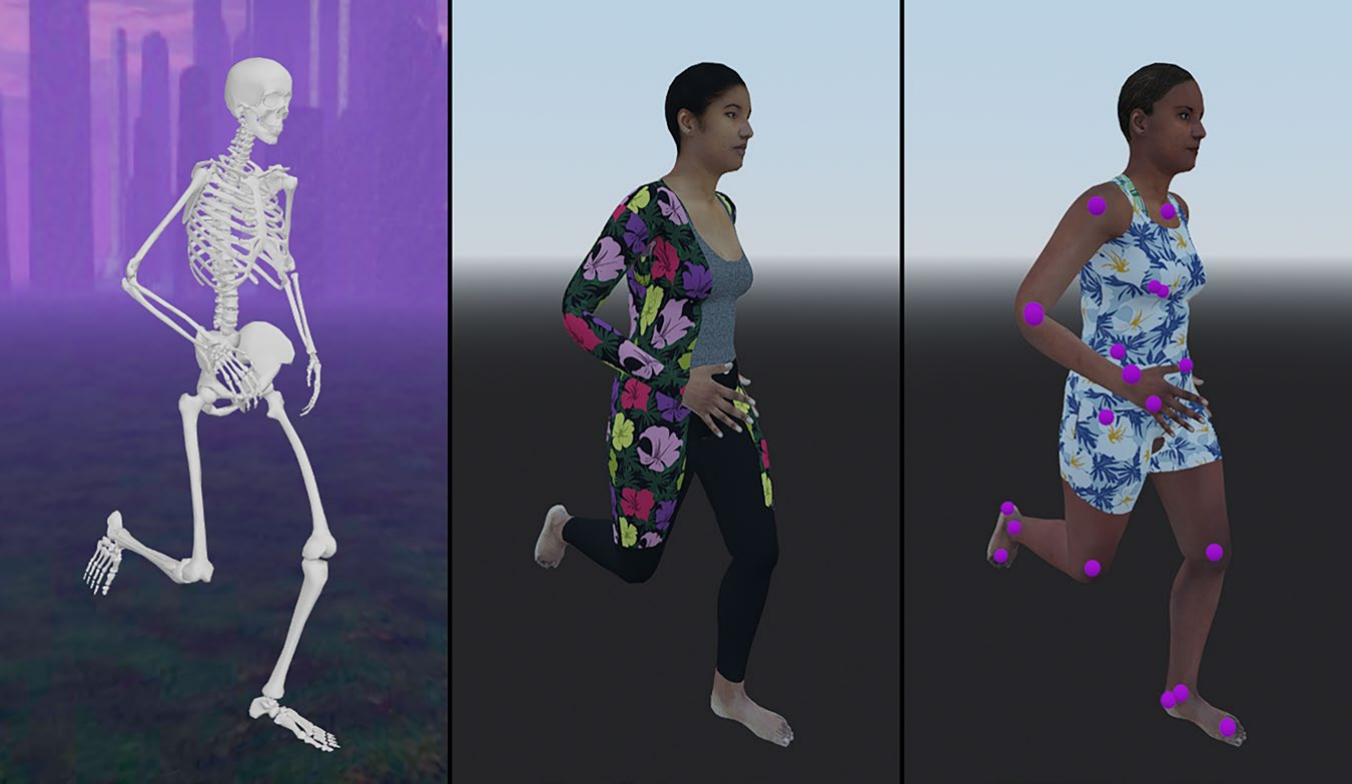
Summary of the workflow to generate the data. We simulated human kinematics with skeletal models (left), associated the motion of skeletal bodies with the deformation of a skin mesh (center), and generated synthetic images of the motion with annotated skeletal landmarks (right).

Our workflow for generating annotated images consists of three separable major steps (Fig. 1):

1. Creating a realistic human mesh with an underlying skeleton from an MSK model for controlling the deformation of the mesh
2. Reconstructing the kinematics of MSK models using the human mesh in a game engine
3. Generating the images, annotations, and labels

## Step 1: Creating realistic human mesh with skeleton-informed deformation

### Creating dimension- and pose-matched skin meshes and musculoskeletal models

In the first step, we started by creating the mesh for our visual avatar of a person. For this purpose, we used the previously developed “Skeletal Kinematics Enveloped by a Learned body model” (SKEL)^26^ to create a skinned multi-person linear model (SMPL)^23^ (https://smpl.is.tue.mpg.de/) based mesh with an underlying MSK model called Biomechanical Skeleton Model (BSM). When given a set of pose and body shape parameters, SKEL generates a mesh of the human skin surface whose morphology and pose depend on the body shape parameters and pose parameters, respectively. These pose parameters are the generalized coordinates of BSM. However, despite being an MSK model, the shoulder joint definition of BSM is incompatible with OpenSim. Therefore, instead of using SKEL directly to generate posed meshes for each frame (i.e., time point) of kinematics, we devised a workaround to enable compatibility with OpenSim.

We wanted our workflow to enable OpenSim-compatible MSK models, so we extracted the translations of the joints of SKEL’s underlying MSK model (i.e., BSM) in T-pose and then scaled and fitted an OpenSim-compatible MSK model (Hamner full-body running model^27^) to the extracted joint translations using OpenSim’s model scaling and inverse kinematics functionalities (Fig. 8, left side). Resultingly, we obtained a skin mesh and an OpenSim-compatible MSK model that were approximately matched in body segment dimensions and pose. What remained next was to associate the pose of the bodies of the MSK model with deformations of the skin mesh, i.e., rigging and skinning the skin mesh.

Note that generating SMPL models posed directly for each kinematics frame is a potential future development that would enable more realistic mesh deformation and potentially anatomically more accurate landmark annotation than our current rigged approach. SKEL already enables this for BSM, but not for OpenSim-compatible models.

### Rigging and skinning the skin mesh

We wanted to use the skeletal structure of the MSK model for creating a skeleton for the skin mesh (i.e., rigging) and define how the displacement of the bones of the created skeleton deforms the skin mesh (i.e., skinning). From the OpenSim-compatible MSK model fitted to the SMPL mesh, we used the OpenSim application programming interface (API) to extract the topology of body segments and joints (i.e., information about which joints the bodies use to connect to each other). Similarly, we extracted the rotations and translational endpoints of body segments in the fitted pose. We then imported the SMPL mesh into the computer graphics software Blender and recreated the skeletal structure of the MSK model by creating a Blender armature with bones whose endpoints were defined by the information we extracted from the MSK model and that were connected and parented according to the topology of the MSK model.

Note that in the context of Blender, we use the term “bone” to refer to rigid objects whose displacement is used to deform associated meshes. Blender bones are created by defining two endpoints, the head and tail of the bone, and by defining if the bone has a parent, and if it does, if it is connected to its parent. A parented bone will retain its transform with respect to the parent bone, meaning it will move with the parent bone. If the parented bone is additionally connected, its head is attached to the tail of the parent bone. Furthermore, the Blender armature we created does not replicate the function of joints of the original MSK model (and does not account for intricacies such as secondary kinematics) but instead recreates the positions and orientations of bodies of the MSK model at a pre-defined pose (in our case, standing T-pose), where the bodies are treated as bones in the armature.

When creating the armature in Blender, we separated bones by their function into three groups: segment bones, control bones, and connector bones.

**Fig. 2:**
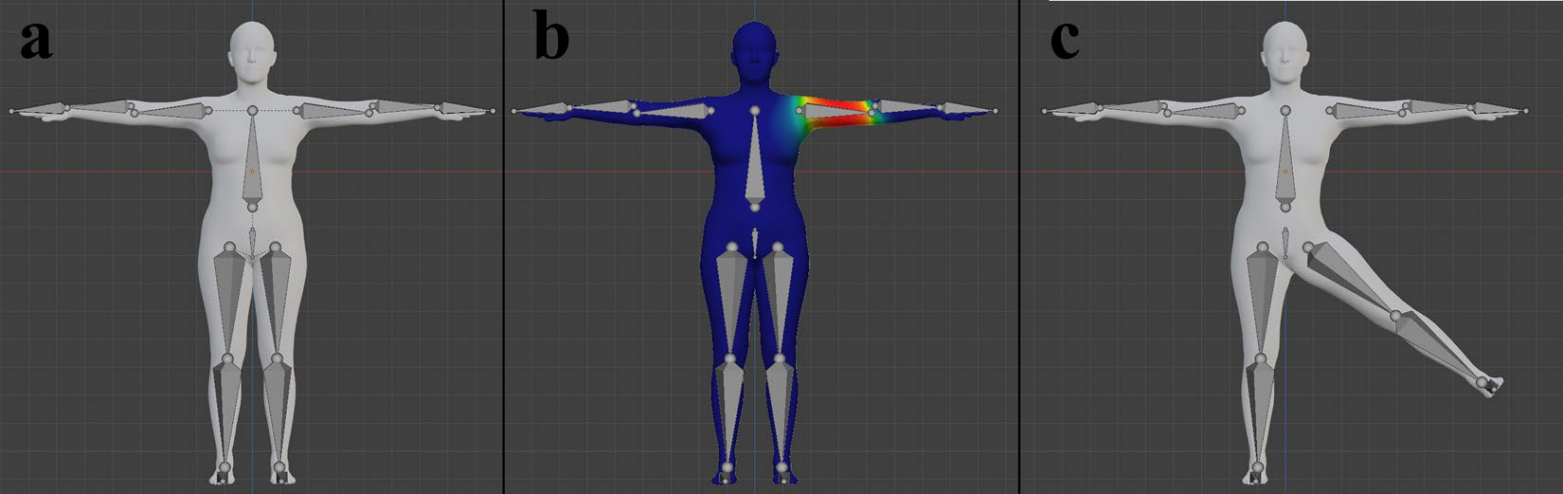
The skeletal structure of the musculoskeletal model was recreated inside the mesh. For illustration purposes, only the segment bones (which correspond to bodies in the musculoskeletal model) are shown. Based on the skeletal structure of the model, we created a Blender armature inside the mesh (**a**) and skinned the mesh to deform with bone motion. Skinning weights were calculated automatically based on the distance between the vertex and the bone (**b**, example showing deformation weights for the left humerus, where red indicates maximal and blue indicates zero deformation). After skinning, the mesh deforms elastically when bones rotate or translate (**c**, example showing mesh deformation with left femur rotation). Note that the segment bones follow the topology of the musculoskeletal model, which is why the bones may connect unrealistically. For instance, the tail of the humerus segment bone and the head of the radius segment bone are not at the same location because the topology defines humerus to connect to the ulna, which in turn connects to the radius; in the figure, the ulna segment bone begins from the tail of the humerus segment bone and ends at the head of the radius segment bone and is too small to be clearly visible.

Segment bones were defined directly according to the endpoints of the skeletal structure of the MSK model. They were used to deform the SMPL mesh by associating each vertex of the mesh with each segment bone with a weight. When a segment bone is moved, the vertices then move with it according to their weights to that segment bone, deforming the mesh (Fig. 2). We calculated the weights automatically based on the distance between the bone and the vertex (Blender’s “bone heat” algorithm).

**Fig. 3:**
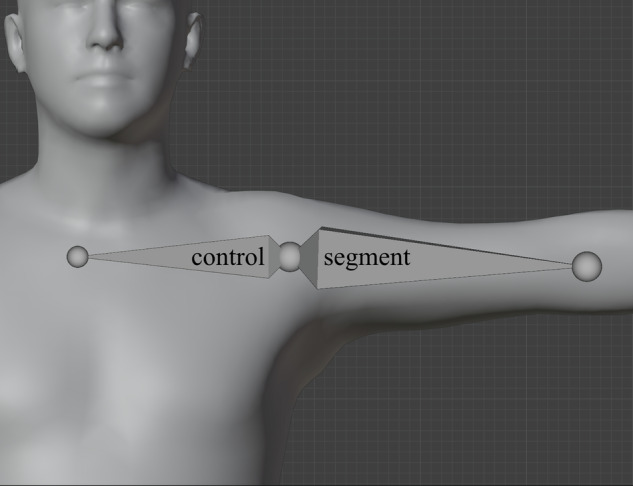
Example of the control bone and the segment bone of the left humerus body segment. The segment bone that controls the deformation of the skin mesh (i.e., the visual avatar) was created according to the skeletal structure of the musculoskeletal model in the fitted pose. The control bone was created as a bone of fixed length starting from the same point as the segment bone, but it was rotated according to the 3D rotation that the left humerus body segment has in the musculoskeletal simulation in this fitted pose – this is why the control bone points in a different direction than the segment bone. When we later set the visual avatar to a different pose in the game engine, we apply the 3D transform of the humerus body segment (obtained from musculoskeletal simulation) to the control bone. The segment bone is parented to the control bone, so it moves with the control bone, and the skin mesh deforms according to the segment bone. If we instead applied the 3D transform of the humerus body segment directly to the segment bone, it would move to an unrealistic position and cause unrealistic deformation of the skin mesh because the segment bone is not informed about the initial rotation (in the fitted pose) of the humerus body segment in the musculoskeletal simulation.

However, applying transforms from OpenSim-simulated kinematics directly to segment bones resulted in unrealistic deformation of the mesh because that method failed to consider the initial transforms that the body segments of the MSK models had in the fitted pose. Hence, we needed to read the transforms of body segments in the fitted pose of the MSK model and incorporate those transforms into the Blender armature. For this purpose, we created a control bone for each segment bone (Fig. 3). The control bones were bones of fixed length that shared their head with and were a parent to a segment bone. The initial rotation of a control bone was set to the rotation of the corresponding body of the MSK model. For instance, the control bone for the right femur was the parent of the right femur segment bone and was rotated according to the rotation of the right femur segment in the MSK model in the fitted pose. Then, by applying MSK kinematics to the control bones, the parented segment bones moved accordingly, while transforming and deforming the mesh as intended.

In cases where the tail of a segment bone and the head of the next segment bone along the kinematic chain were not at the same translational position, we created connector bones to link them. These connector bones had an indirect role in skinning, as explained in the next paragraph.

Lastly, the endpoints of some body segments could not be extracted from the MSK model because those body segments did not connect to a joint further along the kinematic chain (e.g., hands, toes) or they branched to several joints (e.g., torso to shoulders and pelvis to hips). In these cases, defining tails for the corresponding segment bones was difficult, so we defined the bone to continue from its head for a fixed distance (e.g., hands, toes) or set the tail to the midpoint between several connecting joints (e.g., torso to shoulders). For extremities it does not matter if the segment bone is longer or shorter than the part of the mesh corresponding to the body segment, because there will be no vertices of other bodies further along the mesh (if longer) or no segment bones of other bodies further along the kinematics chain (if shorter) to compete for the deformation weights. For branching body segments like the torso, if the tail of the bone is too close to the head of the bone, the segment bone will be small, which will affect its weights to the surrounding vertices. For instance, if the torso segment bone is smaller than the humerus segment bones, the bone heat algorithm may result in the humerus segment bones having a greater effect than the torso on the deformation of the vertices near the armpit of the mesh. To emphasize the underweighted segment bone during skinning, we included connector bones in skinning and then moved their weights to the segment bone from which the connector bone originated.

## Step 2: Reconstructing the kinematics of musculoskeletal models using the human mesh in a game engine

After rigging and skinning the model, we exported it to a game engine. We selected Godot Engine as our game engine of choice because it is free and open-source and because its C +  + module support makes it relatively simple to integrate OpenSim into the project.

### Programmatical implementation

For OpenSim integration into Godot Engine, we wrote a C +  + class called SkeletonTracker. It utilizes the OpenSim API to load stored kinematics (.mot file) and sets the pose of an MSK model (.osim file). Based on the pose of the MSK model, SkeletonTracker extracts global 3D transforms of bodies and joints, virtual marker translations, and values of generalized coordinates (i.e., pelvis translation and joint angles). To access SkeletonTracker with Godot’s in-editor programming language GDScript, we wrote the Godosim class inside a custom Godot module to provide bindings to GDScript. Therefore, SkeletonTracker utilizes the OpenSim API, Godosim utilizes SkeletonTracker, and Godosim can be used inside the Godot Engine editor (see Fig. 8).

#### Godot workflow

In practice, at each frame of the kinematics data, the kinematics of the MSK model extracted by SkeletonTracker is assigned to the rigged and skinned mesh in Godot. Transforms of bodies are assigned to control bones, resulting in the mesh deforming accordingly. Thus, the software can be used to visualize skeletal kinematics with realistic human avatars (as shown in Fig. 1). However, the main purpose of the software is to utilize the visualized kinematics for generating annotated images of motion.

## Step 3: Generating the data, annotations, and labels

In Godot Engine, we created a virtual 3D scene that consists of a camera, the skin mesh, and possible environment and visual effects. The camera is implemented through Godot Engine’s Camera3D node, which tracks the translation, rotation and other parameters of the camera. The camera provides a view into the scene, essentially showing a 2D projection of the 3D scene from the perspective of the camera and enabling the creation of 2D images and annotations of the scene – 3D points in the scene are projected to 2D coordinates using an in-built function of the camera node. The skin mesh is deformed to the pose described by the kinematics at any given frame. During dataset generation, the pose of the skin mesh, camera translation, rotation, field of view (FOV), and other parameters, skin and clothing textures, and backgrounds are changed through different permutations, which ensures that the generated images are richly varied (this protocol is described in more detail further down in “**Dataset generation**”). The technical working principles of the data generation process are described in detail below.

### Color images and keypoint annotations in 2D

**Fig. 4:**
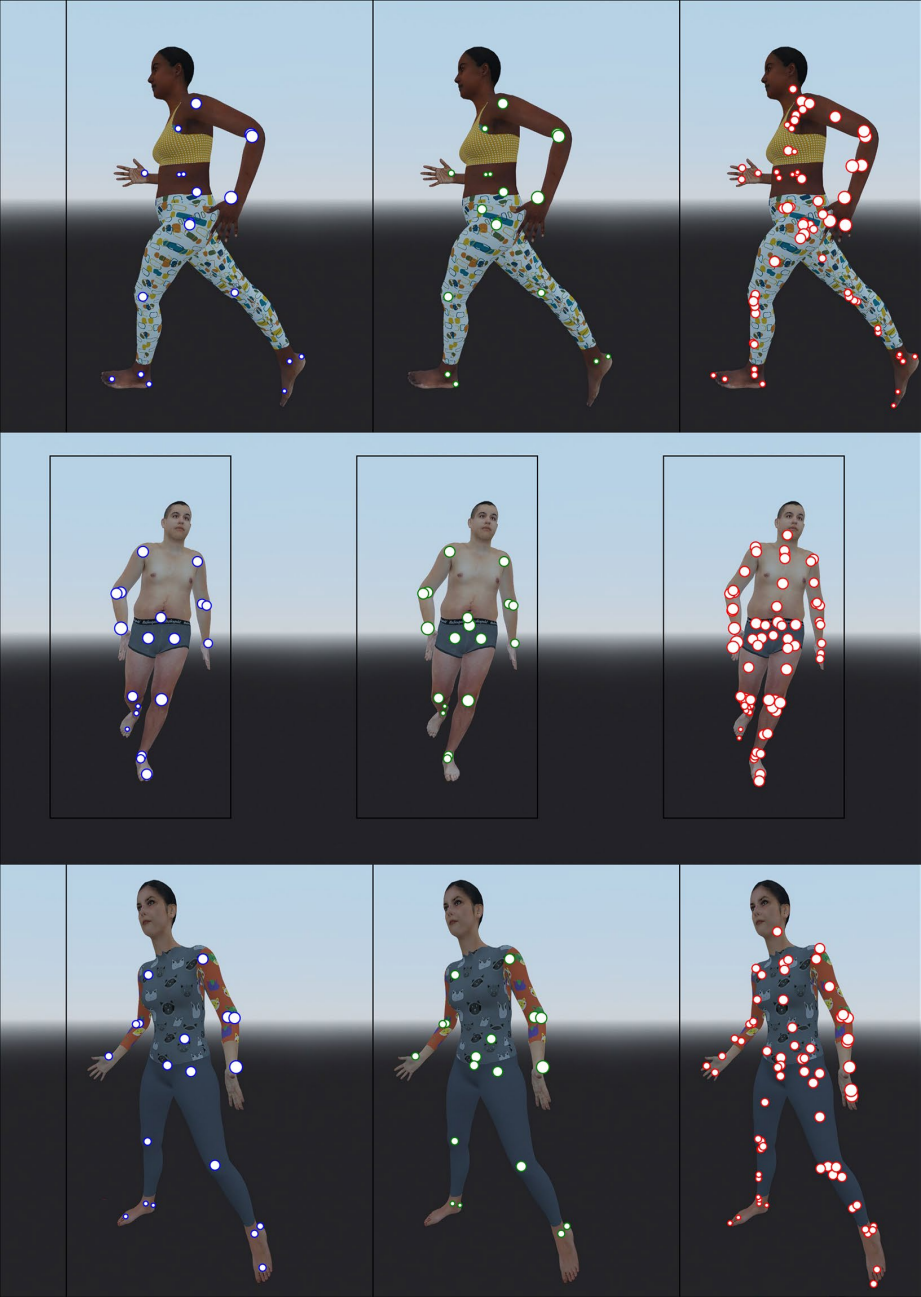
A demonstration of the different keypoint annotations on three visual avatars. The positions of joints, bodies, and virtual markers are visualized by blue, green, and red markers, respectively. The size of the marker indicates its depth, with smaller markers being further away from the camera. Note that the keypoints for joints and bodies largely overlap because joints are often positioned at or near the origin of the connecting body. The bounding box is in black and is fully visible only in the middle row.

When generating data using the software, the camera view is saved as an image at a resolution of 1024 × 1024 pixels. We implement image saving using Godot’s built-in functionality by getting the texture from the current viewport (i.e., the part of the scene that is visible on the display) as an image and saving that image in JPEG format. Additionally, the 3D positions of modelled joints, bodies and virtual markers are projected to the 2D camera view to create ground truth keypoint annotations as pixel coordinates (Fig. 4). In addition to those 2D keypoints of anatomical landmarks, other annotations and labels include 2D bounding boxes enclosing the skin mesh, depth of the keypoints (enabling 3D keypoint coordinates), visibility information of the keypoints, and labels of modelled kinematics.

### Bounding boxes

Bounding boxes are first calculated in 3D by iterating through the vertices of the skin mesh and finding the corners of the smallest 3D box enclosing it. During iteration, along each of the three coordinate axes, if the current position is greater (or smaller) than the currently known maximum (or minimum) boundary, it becomes the new maximum (or minimum) boundary. Because the skin mesh has 7,576 vertices after importing it to Godot Engine and iterating through all of them is computationally cumbersome, we find the corners by iterating only through every 25^th^ vertex. This decision may degrade the resolution of determining the 3D bounding box and may result in some parts of the mesh reaching slightly beyond the bounding box – this effect is examined later in the **Technical Validation** section. To compute the 2D bounding box, the eight corners of the 3D box are projected to the 2D camera view, and the four corners of the resulting 2D bounding box are calculated. The 2D bounding box is enlarged on all four sides by a padding of 25 pixels. We then save the image coordinates of the top-left corner and the width and height of the 2D bounding box in pixels.

### Keypoint depth

The depth of a keypoint is calculated in the 3D scene as the Euclidean distance between the position of the camera and the position of the landmark (e.g., the knee joint) that the keypoint represents. Unlike keypoint annotations, which are given in pixels, keypoint depth is saved in meters. The depth, together with 2D keypoint annotations, constitutes 3D keypoints.

### Visibility labels

Visibility labels are saved as floating-point numbers between 0 and 1, where 0 indicates the least visibility and 1 indicates the best visibility.

To calculate the visibility of any anatomical landmark, we count the number of colliders between the camera and the landmark. In the skin mesh, each body segment is considered a collider. We generate 3D collision shapes for each body segment by iterating through all vertices of the mesh and finding the segment bone with the highest weight for that vertex. The vertex is assigned to belong to the body segment moved by that segment bone. Once each vertex is assigned to a body segment, we use the vertices associated with each body segment to generate 3D collision shapes for the body segments.

**Fig. 5:**
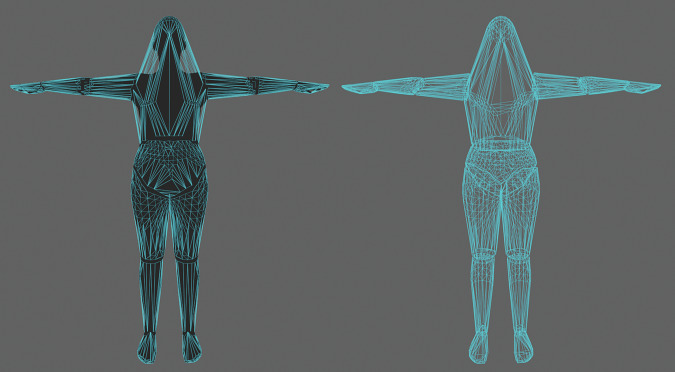
Wireframe representations of the generated collision shapes for body segments with the skin mesh visible (left) and hidden (right). The convexity of the collision shapes results in them failing to follow the skin mesh, particularly around the neck, because the torso and head are considered a single body segment in the musculoskeletal model used in this example.

The collision shapes are implemented using Godot Engine’s ConvexPolygonShape3D node, which does not support concave shapes. Resultingly, some of the collision shapes are inaccurate because they fail to follow the concave surface of the skin mesh (Fig. 5).

Unlike bounding boxes, which are calculated every frame of kinematics data, we calculate and generate collision shapes only once when starting the software, so we can iterate through all vertices without losing much computational performance. Consequently, the generated collision shapes are rigid and only translate and rotate, but do not deform, with changing kinematics. This effect further adds some inconsistency with the skin mesh in poses where the skin mesh is deformed from its original shape.

To count the number of colliders between the camera and the anatomical landmark, we cast a ray from the camera to the 3D location of the landmark using Godot Engine’s RayCast3D node and count how many individual collision shapes the ray intersects before reaching its target. This method yields the number of colliders, which are typically limbs.

Note that the ray is unlikely to reach its target without intersecting at least one collision shape because many anatomical landmarks are located within the collision shape (e.g., joints are inside the body segment, not on its surface). For instance, if the ray intersects the collision shape of the femur segment along its path to the hip joint center, the resulting detected number of colliders is one, even though no other obstacle occludes the hip. However, exceptions may occur if the landmark is situated between two body segments and the ray reaches it at an angle that prevents it from touching the collision shape of either of the body segments, in which case, no colliders are detected.

Finally, to present the visibility label in a more standardized format, we use the equation

$${\rm{visibility}}=\frac{1}{1+N}$$

where *N* is the number of detected colliders. This encodes visibility in a half-open interval (0,1]. A visibility close to zero indicates very low visibility and strong visual occlusion, while a visibility of one indicates no occlusion whatsoever.

In the sample dataset, visibility can be interpreted as a measure of self-occlusion because the images are single-person and the only objects with collision shapes are the body segments of the skin mesh.

### Mask images

**Fig. 6:**
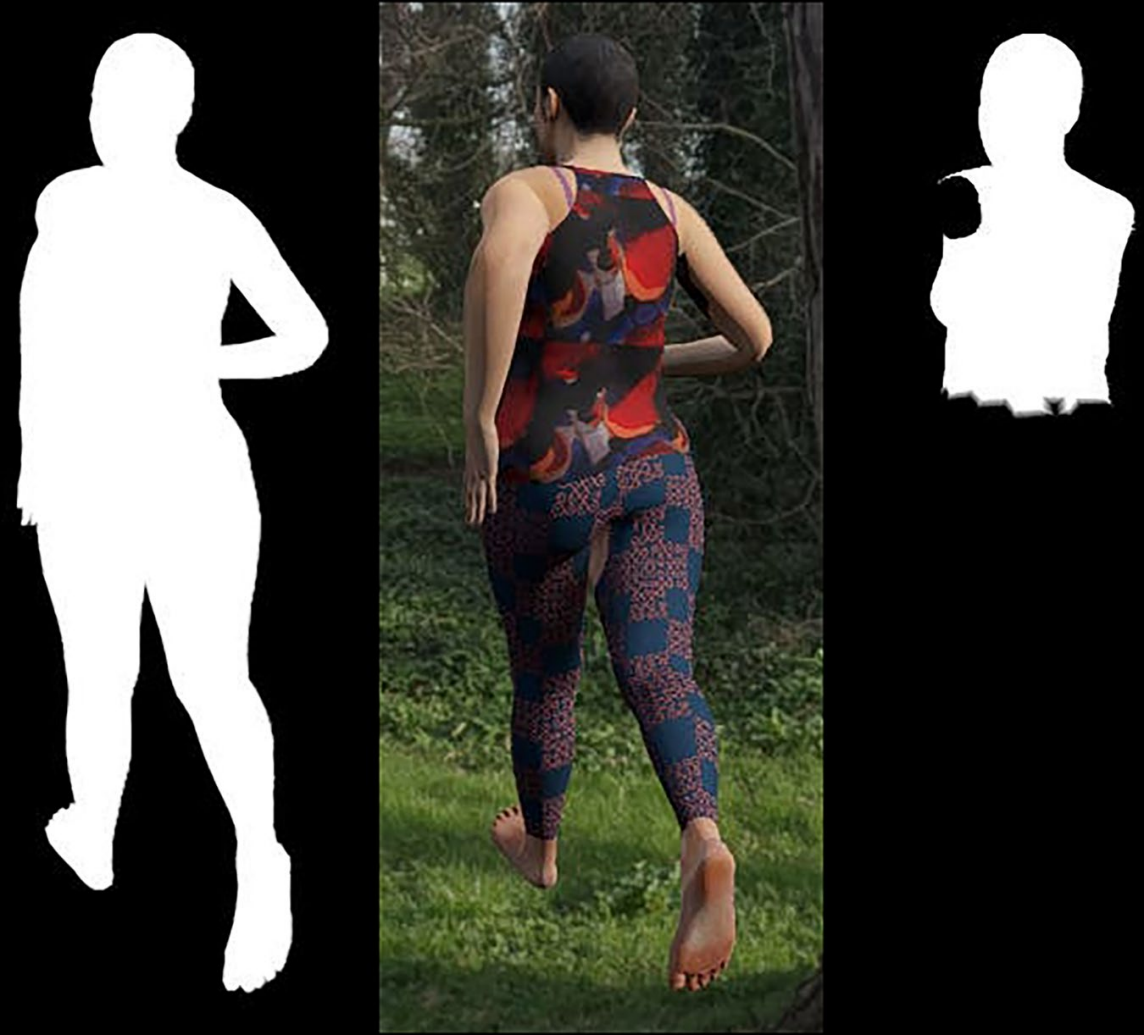
In addition to RGB images (middle), silhouette masks are created of the full skin mesh (left) and its individual body segments (right, segment comprising torso and head as an example).

In addition to the standard RGB image of the camera view, we saved mask images showing the silhouette of the skin mesh and silhouettes of individual segments of the skin mesh. These mask images can be used for training image analysis models for segmentation tasks. When mask images are generated, the background and other visual effects, except for the skin mesh, are excluded from the viewport by switching to another camera object that has a culling mask that only renders the skin mesh. Additionally, we apply a shader to the skin mesh to paint it white. For creating mask images of individual segments, the shader checks each vertex in the skin mesh for weights to bones in the armature and hides vertices whose weight to the bone representing the currently evaluated segment is less than 0.5. The resulting mask images are white areas on a black background (Fig. 6). The utilized musculoskeletal model defines which segments have their own mask image based on which bodies are modelled separately in the musculoskeletal model; in the sample dataset, these segments are the torso (including head), upper arms, lower arms, hands, thighs, shanks, and feet.

### Dataset generation

**Fig. 7:**
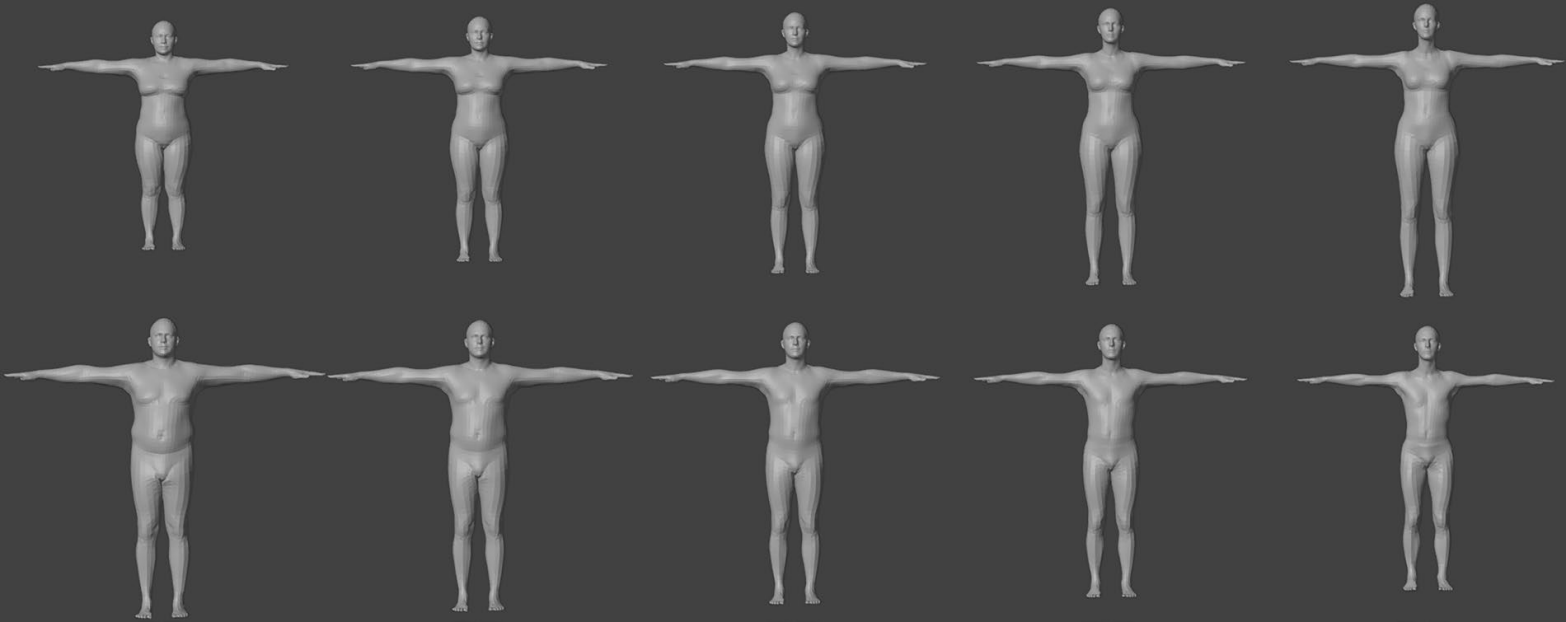
The SKEL-generated SMPL mesh morphologies for females (top row) and males (bottom row) when all ten shape parameters were set to −2, −1, 0, 1, and 2 (columns in order).

After our workflow was developed, we generated a sample dataset of 100,000 images. For the dataset, we generated SMPL meshes using SKEL for ten different body morphologies: male and female variants of five body weights. The SMPL body weight is controlled by ten shape parameters with SKEL recommending a range of values from −2 to 2. For the five different body weights, we set all ten shape parameters to −2, −1, 0, 1, and 2 (Fig. 7).

For further variation of image conditions, we used 50 different skin and 500 different clothing textures from the BEDLAM project^22^ (https://bedlam.is.tue.mpg.de/) and 70 different HDRI backgrounds from Poly Haven (https://polyhaven.com/hdris). We used a variety of randomized camera positions, rotations, and fields of view (FOV between 30 and 120 degrees).

For kinematics (i.e., the poses of the visual avatar), we used a total of 10,000 frames of motion: 5000 frames from 28 trials of recorded motion of various types from an existing motion capture dataset^28^ (https://mocap.cs.sfu.ca/), and 5000 frames from random-generated poses, both explained below. The 28 included real motion trials involved jumping with two feet, walking backwards in a circle, jogging, jump rope exercises, skipping sideways, trotting slowly in a circle, stomping forwards and backwards in a circle, walking in various ways, balancing on one foot, doing cartwheels, crawling, cha-cha dancing, doing yoga, platform jumping, vaulting over obstacles, kendo movements, various rolls, and wushu kicking motions. For each included motion trial in the existing motion capture dataset, we used the marker trajectories in the data for scaling generic MSK models and running inverse kinematics, which yielded the estimated kinematics. From the estimated kinematics, we extracted an equal number of frames to ensure that all the different motions are represented in the final 5000 frames of real motion. Another 5000 frames of motion were obtained by randomly generating kinematics from a uniform distribution within the allowed ranges of values defined in the MSK model. Note that even though we sampled generalized coordinates within allowed joint ranges of motion, many random-generated poses are unrealistic because body segments overlap with other body segments (e.g., the hand reaching inside the torso).

Therefore, the sample dataset is divided into two subsets: “real” (50,000 images, where 5000 frames of kinematics come from real measured motion) and “random” (50,000 images, where 5000 frames of kinematics were generated randomly). The sample dataset is publicly available on Zenodo^29^.

**Fig. 8:**
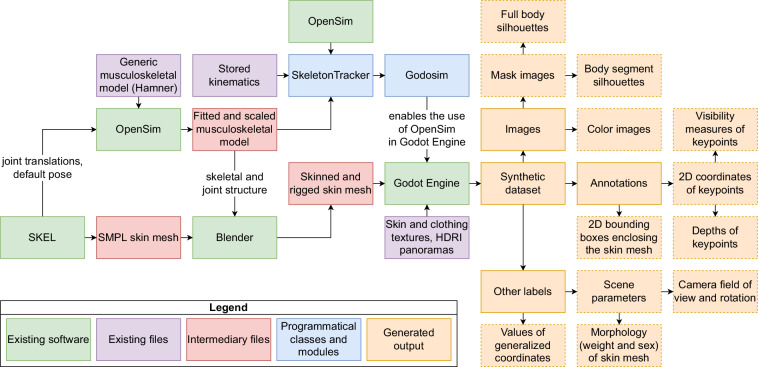
Overview of the workflow and generated data.

A flowchart showing a comprehensive overview of the methods is provided in Fig. 8.

## Data Records

The generated sample dataset of 100,000 annotated images is available on Zenodo^29^. These images require approximately 45 GB of storage space when extracted. The dataset uses SMPL^23^ skin meshes generated using SKEL^26^ with skin and clothing textures from the BEDLAM dataset^22^, and the Hamner full-body running musculoskeletal model^27^ for calculating the poses.

The dataset is organized in ZIP archives of 10,000 images and corresponding annotations, labels, and metadata each. The ZIP archives are named “Output_real_X” or “Output_random_X” for images generated from real human kinematics (analyzed from motion capture data) and random-generated kinematics, respectively. The index X ranges from 1 to 5; the archive with index 1 contains the first 10,000 images, index 2 the second 20,000 images, and so on. Each ZIP archive contains four folders: “annotations”, “images”, “silhouettes”, and “segments”. The “annotations” folder contains a comma-separated value files “annotations.csv”, which contains the annotations and labels of the images, and “metadata.csv”, which contains additional information such as body morphology and camera parameters. The “images” folder contains RGB images of human avatars, while the “silhouettes” folder contains silhouette masks of the avatars, and finally, the “segments” folder contains silhouette masks of individual body segments (see Fig. 6).

## Technical Validation

While the data were generated with the purpose of using them for training HPE models, no such models were trained in this paper. Therefore, the technical validation is focused on (1) assessing the validity of the keypoint annotations in the generated images and (2) demonstrating the diversity of poses and other conditions in the generated sample dataset.

### Validity of generated annotations

We wanted to verify that retrieving the 3D positions of annotations from OpenSim and projecting them to 2D works. For this purpose, we picked the first 10,000 images from the sample dataset and detected keypoints in them using the OpenPose BODY_25 HPE model. When given an image as input, the HPE model detects the 2D positions of shoulder, elbow, wrist, hip, knee, and ankle joints, which are also present in our dataset. Thus, by calculating the Euclidean distances between OpenPose-detected joints and corresponding joint annotations in our dataset, we can verify that the 3D joint locations from OpenSim simulations are correctly transformed into 2D annotations.

**Fig. 9:**
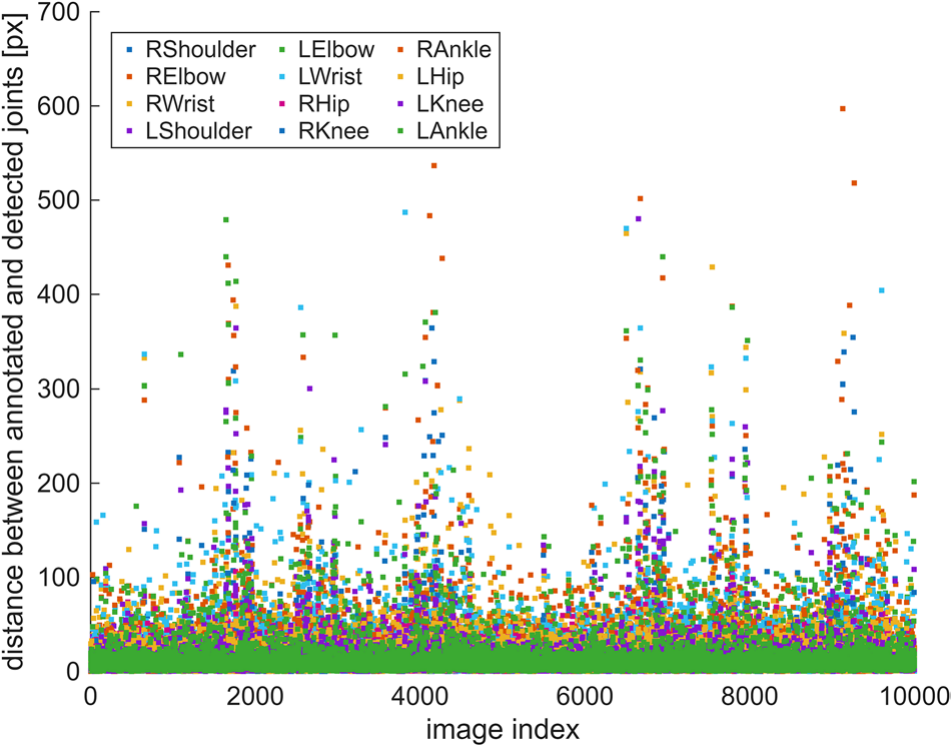
The Euclidean distances in pixels between the positions of various joints detected by an existing HPE model (OpenPose BODY_25) and the positions generated by our method. The original image resolution is 1024 pixels. The data is presented for 10,000 images of the sample dataset and generated from real kinematics. Outlier distances are caused by occlusion-related artifacts in the output of the trained HPE model.

The Euclidean differences show that joints are localized similarly by both OpenPose detections and the annotation procedure of our method (Fig. 9), i.e., generating the annotations appears to work. The outlier points showing great Euclidean distances were caused by OpenPose failing to detect the keypoints accurately mostly because of visual occlusion.

Note that we did not perform an exact comparison between OpenPose-detected keypoints and our annotated positions. Such an analysis in this paper would be meaningless because we would be comparing two different things (annotations in a dataset against the output of a trained HPE model) and because even corresponding joints are localized differently between OpenPose’s training set and our generated dataset. Hence, quantifying measures like mean distance would not enable any proper conclusions on the performance of automatically projecting 3D joint positions from MSK simulations to 2D images. Instead, training HPE models with our data and comparing their output to the output of previous HPE models (such as the BODY_25 model of OpenPose) should be done in a future study.

**Fig. 10:**
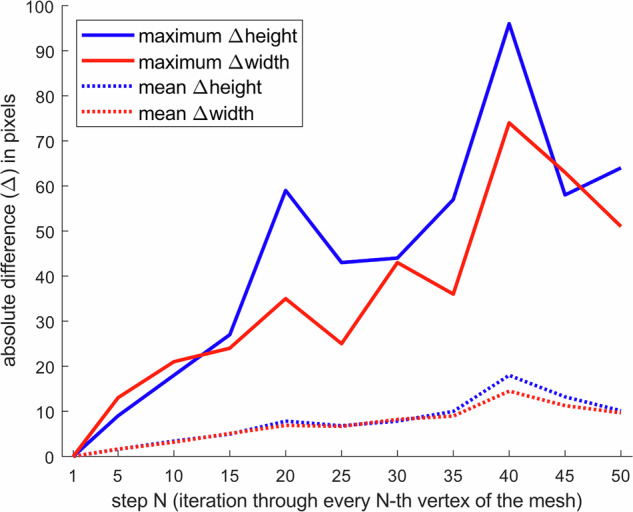
Absolute differences in dimensions (width and height) of generated bounding box annotations for different step parameters N (determining how many vertices are skipped between vertices that are included in calculating the bounding box) compared to iterating through all vertices of the skin mesh (i.e., when the step parameter N = 1).

When generating the bounding boxes, skipping every N-th vertex of the skin mesh may affect the size of the bounding box and result in bounding boxes that do not enclose the skin mesh. To assess this effect, we analyzed how the width and height of the generated bounding boxes changes when we iterate through every 5^th^, 10^th^, 15^th^, 20^th^, 25^th^, 30^th^, 35^th^, 40^th^, 45^th^ and 50^th^ vertex compared with iterating through every single vertex. For this purpose, we generated a set of 100 images of random poses with a similar protocol to that of the sample dataset. Analysis of the bounding box dimensions showed that while the differences in bounding box dimensions mostly remain below 20 pixels when the step parameter is up to 50, the difference in bounding box dimensions in outlier cases can be almost 100 pixels (Fig. 10). Step parameters of 20 and 40 likely result in skipping vertices that are important for defining the bounding box, such as those at the ends of extremities like the head, hands, or feet. Therefore, our choice of step parameter 25 and padding of 30 in each direction should suffice in the sample dataset.

While the bounding boxes in the sample dataset are generated iterating through every 25^th^ vertex, users of the software may define the step parameter and boundary box padding amount manually if they generate their own images. Instead of iterating through every N-th vertex, users may alternatively use a small pre-defined set of vertex indices that are configured for the SMPL mesh – this may be a faster solution when using SMPL meshes; instructions to do so are provided in the GitHub repository.

### Distribution of kinematics in the sample dataset

**Fig. 11:**
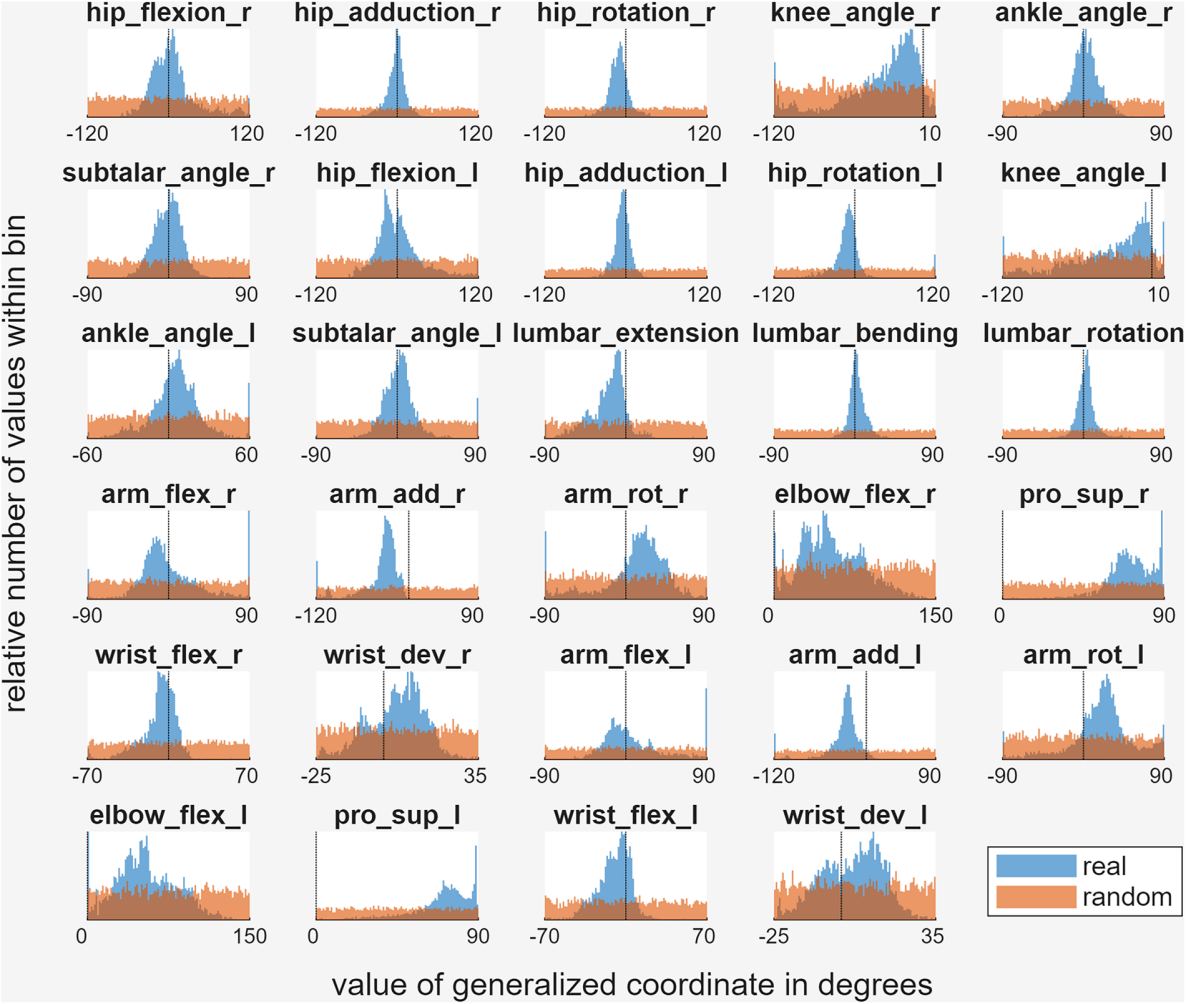
The distributions of the values of the generalized coordinates of the musculoskeletal model in the published dataset, where the kinematics were solved from real measured motion (blue) and generated randomly (orange). The different subplots show the distributions for different generalized coordinates defined in the musculoskeletal model and are bound to the valid range of that generalized coordinate. Black dashed lines show where the value of the generalized coordinate is zero.

The relative distribution of MSK generalized coordinates is shown in Fig. 11 for kinematics of real motion analyzed from an existing motion capture dataset^28^ (shown in blue) and randomized motion (shown in orange). The randomized kinematics are unsurprisingly distributed much more uniformly than the kinematics from real measured motion, where certain poses naturally occur more frequently than others. This is an important observation if the data is used for training models for estimating the values of generalized coordinates, because the relatively higher presence of certain values may introduce bias towards outputting those values when using the trained models. To account for this limitation, rather than using all the data as is, annotated training images with uncommon kinematics could be weighted more heavily, or some annotated training images with commonly occurring kinematics could be omitted when the data is used for training such models.

## Usage Notes

We share our work in three levels of increasing customizability. First, we have generated a sample dataset of 100,000 annotated images, which is available on Zenodo^29^ (as described in the **Data Records** section). These images should provide a usable and demonstrative example of the functionality of the software. If the user simply wishes to use the data, downloading the sample dataset is the easiest way.

Second, we provide a binary application for running the software and generating synthetic photos, pre-built for Windows and available on GitHub (https://github.com/jerela/Godosim). The pre-built application allows limited customization through pre-set photoshoot settings that define, e.g., where the camera is placed. The user can also plug in different human skin meshes (e.g., if they have a more accurate mapping from the skeletal model to the skin mesh), change textures and backgrounds, or change the annotated keypoints by modifying the virtual marker set in the musculoskeletal model.

Third, we share the source code of our software on GitHub (https://github.com/jerela/Godosim), which provides full customizability over the workflow. Using the source code requires recompiling Godot Engine and building the software application, so it is only recommended for experienced users.

The instructions for the second and third options are provided in detail in our GitHub repository. The repository also contains a Python script for overlaying the annotations on the images for illustration purposes. In this paper, we describe the overall workflow and give the reader the information they need to use the sample dataset.

In the dataset, we used SMPL meshes and textures. However, the workflow could be used for other skin meshes and textures as well. To do so, step 1 of methods would have to be adapted for the different skin mesh, but step 2 would remain identical. Because we use textures from another project, we cannot share the textures and those must be downloaded from the original source. The instructions to do so are provided in the GitHub repository.

## Data Availability

The generated data is available on Zenodo (https://zenodo.org/records/15525581)^29^.

## Code availability

A repository with the code is available on GitHub (https://github.com/jerela/Godosim). Detailed installation instructions are provided in the repository.
